# *p*-Phenyl­enedimethanaminium dibromide

**DOI:** 10.1107/S1600536810024840

**Published:** 2010-06-30

**Authors:** Yuan Zhang, Meng Ting Han

**Affiliations:** a Ordered Matter Science Research Center, College of Chemistry and Chemical Engineering, Southeast University, Nanjing 211189, People’s Republic of China

## Abstract

In the title salt, C_8_H_14_N_2_^2+^·2Br^−^, the cation has a crystallographically imposed centre of symmetry. The compound is isostructural with the chloride analogue. In the crystal structure, the cations and anions are connected *via* N—H⋯Br hydrogen bonds, forming layers parallel to the *bc* plane.

## Related literature

For the synthesis, structures and properties of ferroelectric organic or inorganic compounds, see: Haertling (1999 ▶); Homes *et al.* (2001 ▶); Fu *et al.* (2009 ▶); Hang *et al.* (2009 ▶). For the structure of the isostructural chloride salt, see: Arkenbout *et al.* (2007 ▶).
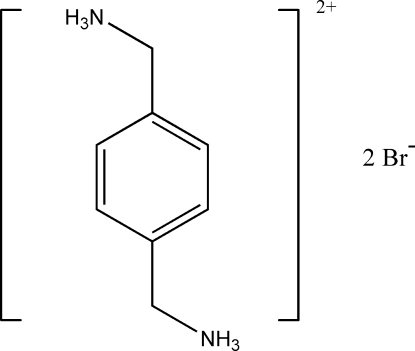


## Experimental

### 

#### Crystal data

C_8_H_14_N_2_^2+^·2Br^−^
*M* *_r_* = 298.01
Triclinic, 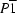

*a* = 4.4462 (9) Å
*b* = 6.0331 (12) Å
*c* = 10.347 (2) Å
α = 101.90 (3)°
β = 99.79 (3)°
γ = 94.29 (3)°
*V* = 265.89 (9) Å^3^
*Z* = 1
Mo *K*α radiation
μ = 7.58 mm^−1^
*T* = 293 K
0.20 × 0.20 × 0.20 mm

#### Data collection

Rigaku Mercury2 diffractometer
Absorption correction: multi-scan (*CrystalClear*; Rigaku, 2005 ▶) *T*_min_ = 0.837, *T*_max_ = 1.000
2767 measured reflections
1213 independent reflections
1107 reflections with *I* > 2σ(*I*)
*R*_int_ = 0.053

#### Refinement

*R*[*F* ^2^ > 2σ(*F* ^2^)] = 0.034
*wR*(*F* ^2^) = 0.084
*S* = 1.10
1213 reflections
56 parameters
H-atom parameters constrained
Δρ_max_ = 0.59 e Å^−3^
Δρ_min_ = −0.68 e Å^−3^

### 

Data collection: *CrystalClear* (Rigaku, 2005 ▶); cell refinement: *CrystalClear*; data reduction: *CrystalClear*; program(s) used to solve structure: *SHELXS97* (Sheldrick, 2008 ▶); program(s) used to refine structure: *SHELXL97* (Sheldrick, 2008 ▶); molecular graphics: *SHELXTL/PC* (Sheldrick, 2008 ▶); software used to prepare material for publication: *SHELXTL/PC*.

## Supplementary Material

Crystal structure: contains datablocks I, global. DOI: 10.1107/S1600536810024840/rz2469sup1.cif

Structure factors: contains datablocks I. DOI: 10.1107/S1600536810024840/rz2469Isup2.hkl

Additional supplementary materials:  crystallographic information; 3D view; checkCIF report

## Figures and Tables

**Table 1:** Hydrogen-bond geometry (Å, °)

*D*—H⋯*A*	*D*—H	H⋯*A*	*D*⋯*A*	*D*—H⋯*A*
N1—H1*A*⋯Br1^i^	0.89	2.49	3.359 (3)	167
N1—H1*B*⋯Br1^ii^	0.88	2.59	3.363 (3)	146
N1—H1*C*⋯Br1^iii^	0.89	2.55	3.422 (3)	167

Symmetry codes: (i) 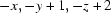; (ii) 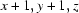; (iii) 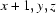.

**Fig. 1.**
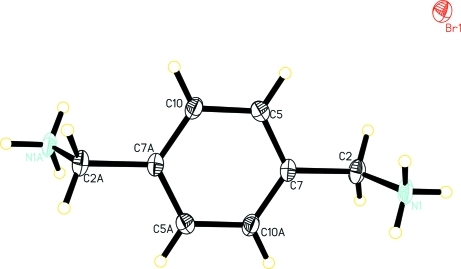
The structure of the title compound showing the atom-numbering scheme. Displacement ellipsoids are drawn at the 30% probability level. Atoms labelled with the suffix A are related by the symmetry code (1-x, 1-y, 1-z).

**Fig. 2.**
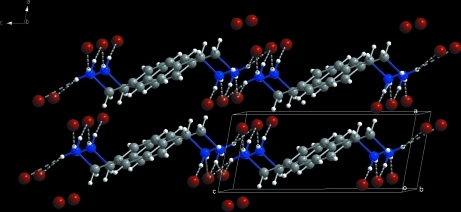
Crystal packing of the title compound approximately viewed along the b axis, showing the hydrogen bondings network.

## supplementary crystallographic information

### Comment

At present, much attention in ferroelectric material field is focused on developing ferroelectric pure organic or inorganic compounds (Haertling, 1999; Homes *et al.*, 2001). Recently we have reported the synthesis of a variety of compounds (Fu *et al.*, 2009; Hang *et al.*, 2009), which have potential piezoelectric and ferroelectric properties. In order to develop new materials of this kind, we investigated the physical properties of the title compound. Its dielectric constant as a function of temperature (93-440 K) indicates that the permittivity is basically temperature-independent (dielectric constant ranging from 3.2 to 4.4), suggesting that this compound should be not a real ferroelectrics or that no distinct phase transition occurred within the measured temperature range. Herein, we report the synthesis and crystal structure of the title compound (Fig. 1).

The cation of the title compound possesses crystallographically imposed centre of symmetry. The compound is isostructural to the chloride analogue (Arkenbout *et al.*, 2007). Bond lengths and angles are within their normal ranges. In the crystal packing (Fig. 2), cations and anions are connected *via* intermolecular N—H···Br hydrogen bonds (Table 1) to form layers parallel to the *bc* plane. Contrary to what observed in the chloride salt, the separation between the centroids of stacked aromatic rings (4.446 (2) Å) suggests that no π···π stacking interactions are present.

### Experimental

Hydrobromic acid (4.05 g, 40%) was added slowly to a solution of 1,4-phenylenedimethanamine (2.72 g, 0.02 mol) in methanol. After several days, colourless prismatic crystals of the title compound suitable for X-ray analysis were obtained on slow evaporation of the solvent.

### Refinement

H atoms were positioned geometrically and refined using a riding model, with C—H = 0.93-0.97 Å, N—H = 0.89 Å, and with *U*_iso_(H) = 1.2_eq_(C, N).

### Crystal data

**Table:**

C_8_H_14_N_2_^2+^·2Br^−^	*Z* = 1
*M**_r_* = 298.01	*F*(000) = 146
Triclinic, *P*1	*D*_x_ = 1.861 Mg m^−^^3^
Hall symbol: -P 1	Mo *K*α radiation, λ = 0.71073 Å
*a* = 4.4462 (9) Å	Cell parameters from 1213 reflections
*b* = 6.0331 (12) Å	θ = 2.6–27.5°
*c* = 10.347 (2) Å	µ = 7.58 mm^−^^1^
α = 101.90 (3)°	*T* = 293 K
β = 99.79 (3)°	Prism, colorless
γ = 94.29 (3)°	0.20 × 0.20 × 0.20 mm
*V* = 265.89 (9) Å^3^	

### Data collection

**Table:**

Rigaku Mercury2 diffractometer	1213 independent reflections
Radiation source: fine-focus sealed tube	1107 reflections with *I* > 2σ(*I*)
graphite	*R*_int_ = 0.053
Detector resolution: 13.6612 pixels mm^-1^	θ_max_ = 27.5°, θ_min_ = 3.5°
CCD_Profile_fitting scans	*h* = −5→5
Absorption correction: multi-scan (*CrystalClear*; Rigaku, 2005)	*k* = −7→7
*T*_min_ = 0.837, *T*_max_ = 1.000	*l* = −13→13
2767 measured reflections	

### Refinement

**Table:**

Refinement on *F*^2^	Secondary atom site location: difference Fourier map
Least-squares matrix: full	Hydrogen site location: inferred from neighbouring sites
*R*[*F*^2^ > 2σ(*F*^2^)] = 0.034	H-atom parameters constrained
*wR*(*F*^2^) = 0.084	*w* = 1/[σ^2^(*F*_o_^2^) + (0.0314*P*)^2^] where *P* = (*F*_o_^2^ + 2*F*_c_^2^)/3
*S* = 1.10	(Δ/σ)_max_ < 0.001
1213 reflections	Δρ_max_ = 0.59 e Å^−^^3^
56 parameters	Δρ_min_ = −0.68 e Å^−^^3^
0 restraints	Extinction correction: *SHELXL97* (Sheldrick, 2008), Fc^*^=kFc[1+0.001xFc^2^λ^3^/sin(2θ)]^-1/4^
Primary atom site location: structure-invariant direct methods	Extinction coefficient: 0.102 (8)

### Special details

**Table:**

Geometry. All e.s.d.'s (except the e.s.d. in the dihedral angle between two l.s. planes) are estimated using the full covariance matrix. The cell e.s.d.'s are taken into account individually in the estimation of e.s.d.'s in distances, angles and torsion angles; correlations between e.s.d.'s in cell parameters are only used when they are defined by crystal symmetry. An approximate (isotropic) treatment of cell e.s.d.'s is used for estimating e.s.d.'s involving l.s. planes.
Refinement. Refinement of *F*^2^ against ALL reflections. The weighted *R*-factor *wR* and goodness of fit *S* are based on *F*^2^, conventional *R*-factors *R* are based on *F*, with *F* set to zero for negative *F*^2^. The threshold expression of *F*^2^ > σ(*F*^2^) is used only for calculating *R*-factors(gt) *etc*. and is not relevant to the choice of reflections for refinement. *R*-factors based on *F*^2^ are statistically about twice as large as those based on *F*, and *R*- factors based on ALL data will be even larger.

### Fractional atomic coordinates and isotropic or equivalent isotropic displacement parameters (Å^2^)

**Table:**

	*x*	*y*	*z*	*U*_iso_*/*U*_eq_	
N1	0.5050 (7)	0.6880 (4)	0.8667 (3)	0.0352 (6)	
H1A	0.4270	0.7394	0.9393	0.042*	
H1B	0.6628	0.7845	0.8646	0.042*	
H1C	0.5668	0.5525	0.8698	0.042*	
C2	0.2679 (7)	0.6652 (6)	0.7437 (3)	0.0354 (8)	
H2D	0.1943	0.8121	0.7417	0.042*	
H2E	0.0952	0.5593	0.7466	0.042*	
C5	0.3474 (8)	0.3493 (5)	0.5579 (3)	0.0345 (7)	
H5A	0.2447	0.2476	0.5965	0.041*	
C7	0.3911 (7)	0.5816 (5)	0.6174 (3)	0.0293 (7)	
C10	0.4567 (8)	0.2702 (6)	0.4418 (3)	0.0353 (8)	
H10A	0.4276	0.1150	0.4028	0.042*	
Br1	−0.14338 (7)	0.20342 (5)	0.87105 (3)	0.03667 (19)	

### Atomic displacement parameters (Å^2^)

**Table:**

	*U*^11^	*U*^22^	*U*^33^	*U*^12^	*U*^13^	*U*^23^
N1	0.0523 (17)	0.0363 (15)	0.0199 (14)	0.0134 (12)	0.0156 (11)	0.0027 (12)
C2	0.0390 (17)	0.0412 (19)	0.0265 (17)	0.0107 (14)	0.0140 (13)	0.0005 (15)
C5	0.0451 (18)	0.0314 (17)	0.0300 (19)	0.0020 (13)	0.0145 (14)	0.0088 (15)
C7	0.0343 (16)	0.0345 (17)	0.0197 (16)	0.0071 (12)	0.0074 (12)	0.0040 (14)
C10	0.055 (2)	0.0265 (16)	0.0249 (18)	0.0067 (14)	0.0131 (15)	0.0013 (14)
Br1	0.0478 (3)	0.0329 (3)	0.0303 (3)	0.00763 (15)	0.01254 (16)	0.00384 (18)

### Geometric parameters (Å, °)

**Table:**

N1—C2	1.483 (4)	C5—C10	1.380 (4)
N1—H1A	0.8880	C5—C7	1.394 (4)
N1—H1B	0.8839	C5—H5A	0.9300
N1—H1C	0.8865	C7—C10^i^	1.382 (5)
C2—C7	1.509 (4)	C10—C7^i^	1.382 (5)
C2—H2D	0.9700	C10—H10A	0.9300
C2—H2E	0.9700		
			
C2—N1—H1A	109.9	H2D—C2—H2E	107.9
C2—N1—H1B	109.6	C10—C5—C7	120.0 (3)
H1A—N1—H1B	109.3	C10—C5—H5A	120.0
C2—N1—H1C	109.0	C7—C5—H5A	120.0
H1A—N1—H1C	109.4	C10^i^—C7—C5	119.1 (3)
H1B—N1—H1C	109.6	C10^i^—C7—C2	121.7 (3)
N1—C2—C7	111.9 (2)	C5—C7—C2	119.3 (3)
N1—C2—H2D	109.2	C5—C10—C7^i^	120.9 (3)
C7—C2—H2D	109.2	C5—C10—H10A	119.5
N1—C2—H2E	109.2	C7^i^—C10—H10A	119.5
C7—C2—H2E	109.2		

Symmetry codes: (i) −*x*+1, −*y*+1, −*z*+1.

### Hydrogen-bond geometry (Å, °)

**Table:**

*D*—H···*A*	*D*—H	H···*A*	*D*···*A*	*D*—H···*A*
N1—H1A···Br1^ii^	0.89	2.49	3.359 (3)	167
N1—H1B···Br1^iii^	0.88	2.59	3.363 (3)	146
N1—H1C···Br1^iv^	0.89	2.55	3.422 (3)	167

Symmetry codes: (ii) −*x*, −*y*+1, −*z*+2; (iii) *x*+1, *y*+1, *z*; (iv) *x*+1, *y*, *z*.
